# Bibliometric analysis of functional dyspepsia research trends over the past 20 years

**DOI:** 10.3389/fpubh.2022.1019110

**Published:** 2022-11-23

**Authors:** Xinai Wang, Hao Liu, Wenjing Li, Hongling Xiao

**Affiliations:** ^1^The School of Graduate, Tianjin University of Traditional Chinese Medicine, Tianjin, China; ^2^The School of Nursing, Tianjin University of Traditional Chinese Medicine, Tianjin, China

**Keywords:** functional dyspepsia, health care, anxiety, COVID-19, bibliometric analysis

## Abstract

**Background:**

Functional dyspepsia is one of the most common functional gastrointestinal disorders that affects the physical health and quality of life of many people. Its status as a chronic disease has received continued attention in the field of gastrointestinal research.

**Objective:**

Bibliometric methods using network analysis were used to identify developments and research trends in functional dyspepsia with a view to informing and orienting further in-depth research on functional dyspepsia.

**Method:**

Bibliometric methods were used to analyze the documents on functional dyspepsia published from 2002 to 2022 retrieved from Web of Science Core Collection on 1 July 2022, import literature data into Excel and VOSviewer, and extract relevant information to analyze and visualize the annual number of publications, authors, organizations, countries, journals published, citations, and keywords.

**Results:**

A total of 3,532 documents were retrieved, and the abstracts of each remaining documents were read one by one after four duplicate documents were removed, and 2,220 documents were included after screening, with a fluctuating growth trend. Tack J ranked first with 171 documents, followed by Talley NJ (*n* = 167). The top three organizations in terms of number of publications were Katholieke Universiteit Leuven (*n* = 131), Mayo Clinic (*n* = 127), and the University of Newcastle (n = 91). The most prolific country was the United States with 499 documents. The three journals with the highest number of publications are “Neurogastroenterology and Motility” (*n* = 218), “Alimentary pharmacology & therapeutics” (*n* = 101), and “Journal of Gastroenterology and Hepatology” (*n* = 90). The top three most cited documents were “Functional gastroduodenal disorders,” “Childhood Functional Gastrointestinal Disorders: Child/Adolescent,” and “The Serotonin Signaling System: From Basic Understanding to Drug Development for Functional GI Disorders.” Frequency counts and network co-occurrences of keywords reveal trends in this field, including “gastric emptying,” “anxiety,” “acupuncture,” and “ghrelin.”

**Conclusion:**

The study of the mechanism of gut–brain interaction in functional dyspepsia and the combination of non-pharmacological treatment and pharmacological treatment may be the future research hotspots and trends. Our findings are helpful to comprehensively review the research history of FD and provide reference for researchers in this field to further study.

## Introduction

Functional gastrointestinal disease as a chronic disease is a very common clinical condition encountered in the clinical practice of gastroenterology, and the Rome IV redefines functional gastrointestinal disease as disorders of gut–brain interaction (1). Functional dyspepsia (FD) is one of its most common types and is divided into two subtypes, postprandial distress syndrome and epigastric pain syndrome, with a global prevalence of 10–30% and regional variations in prevalence (2–4). Its pathogenesis has not been fully elucidated so far, and it is generally believed to be related to a variety of pathophysiological mechanisms, with existing etiologies, including gastrointestinal motility disorders, visceral hypersensitivity, altered gastrointestinal microbiota, immune dysfunction, psychosocial factors, and genetic susceptibility (5, 6). Due to the complex pathogenesis, treatment with gastrointestinal stimulants, antacids, and antidepressants is often used clinically, which is effective but has varying degrees of side effects and a high relapse rate after discontinuation of medication (7). As a non-organic disease, FD has a high prevalence and persistent symptoms that seriously affect the health and quality of life of patients and increase their personal and family healthcare costs (8). In addition, the incidence and recurrence rates of the disease have increased significantly under the influence of COVID-19, which consumes a large number of medical resources and places a heavy burden on the healthcare system (9, 10). As a serious public health problem, it is urgent to find effective treatment options and raise public awareness of it.

Bibliometrics has been applied as a statistical tool in several fields, and it can analyze the literature in various fields qualitatively or quantitatively and further predict the trend of the field (11–13). This approach is important for understanding the content of research in each field. The use of bibliometric methods allows the analysis of authors, organizations, countries, journals, cited literature, and cited references in the field. In addition, the visual analysis of keywords can help us to deeply understand the research hotspots in this field and predict the future development trend. As the field of gastrointestinal research is further developed, the research topics of FD are becoming more and more diverse and involve a wider range of people, and there is an urgent need for a quantitative analysis of its research atlas to understand the core team and the current status and development trend of research in this field (14). However, no bibliometric analysis of the FD research area is available to date. In this study, we will use Vosviewer software based on Java environment to analyze and explore the current status of FD research in the last 20 years. Through the retrospective analysis of the research field of FD in recent 20 years, we hope to help clinicians have a clearer understanding of this field and provide reference for future researchers.

## Methods

### Data sources and search strategy

All literature data were retrieved from the Web of Science (WOS) Core Collection *via* the Tianjin University of Traditional Chinese Medicine Library website on 1 July 2022, using the following search terms: (functional dyspepsia OR non-organic dyspepsia OR non-ulcerative dyspepsia). The publication dates considered in this study were from 1 July 2002 to 1 July 2022, and the types of publications included were reviews or articles published in English. Exclusion criteria were duplicate articles, early online publications, incomplete articles, meeting summaries, and articles not related to this topic. The retrieved articles are exported and saved as plain text files and stored in savedrecs.txt format.

### Data acquisition and cleaning

VOSviewer is a science metrics network analysis software developed by the Center for Science and Technology Research at Leiden University in the Netherlands (15). It can analyze authors, organizations, countries, and citations and visualize keywords to discover hotspots in that research area and predict future trends. In this study, all literature data from the WOS Core Collection were imported into Excel 2016 and VOSviewer v.1.6.18. Before using VOSviewer to analyze the data, the incorporated data were cleaned and sorted out. Some keywords with the same meaning, such as (“functional dyspepsia” and “FD”) and (“functional gastrointestinal disorders” and “FGIDs”), were combined into one term. In addition, synonyms are also combined, for example (“Rome” and “Rome criteria”). The same process is implemented for authors, organizations, and countries. Finally, the cleaned and inspected data were imported into VOSviewer for correlation analysis.

### Data analysis

Each study in VOSviewer is represented by a circle whose diameter size indicates the frequency of occurrence, link strength, or total link strength. The line between two terms indicates whether they are related or not, and the thickness of the line indicates the degree of association. The studied contents are automatically grouped and clustered using different colors and the number of clusters can vary depending on the similarity threshold between nodes. The parameters of the VOSviewer were set as follows: Method (Fractionalization or Association strength). Layout values are set differently depending on the needs of the graphic.

## Results

### Annual publication

**Figure 1:**
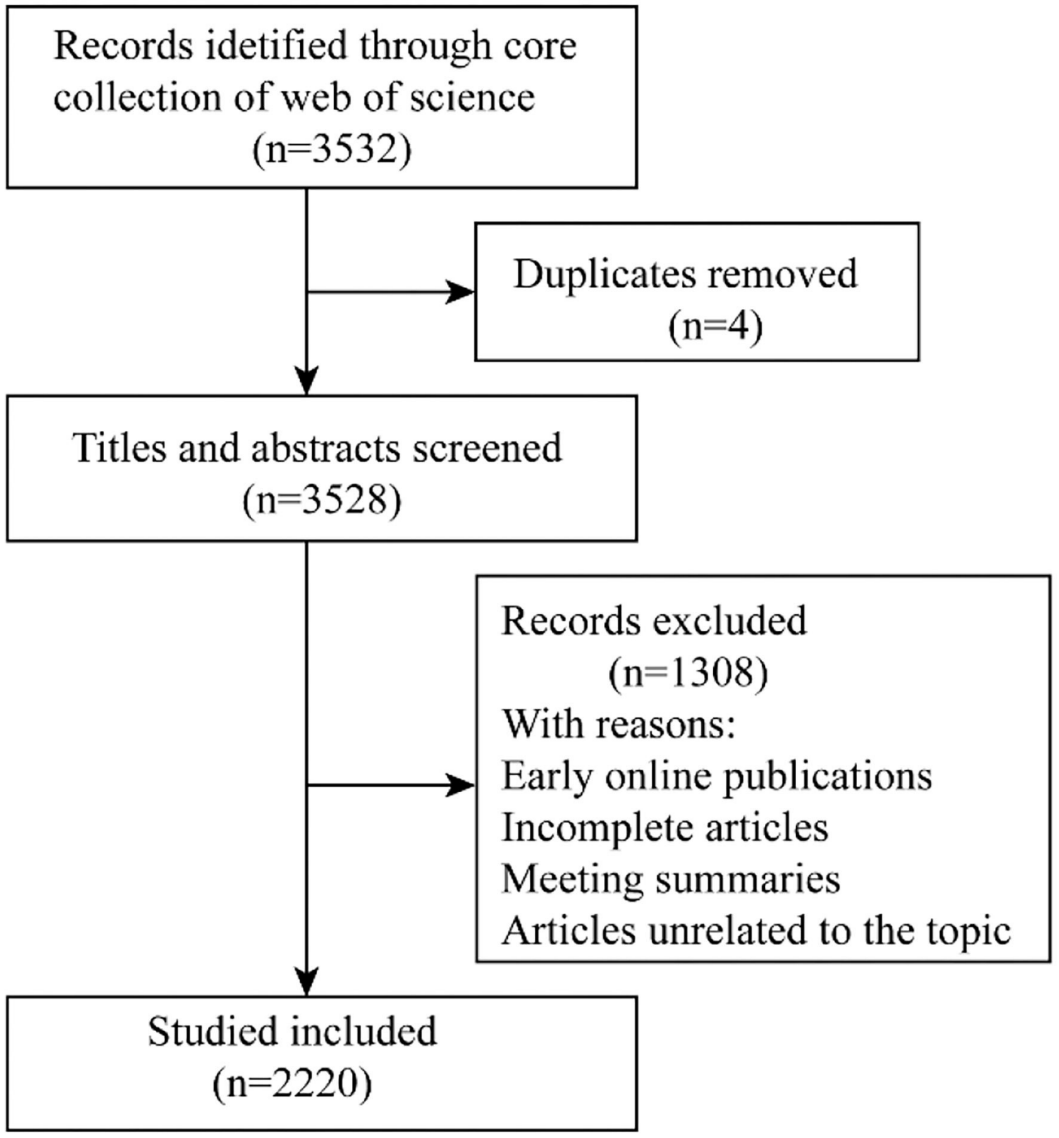
Flow diagram of study selection.

**Figure 2:**
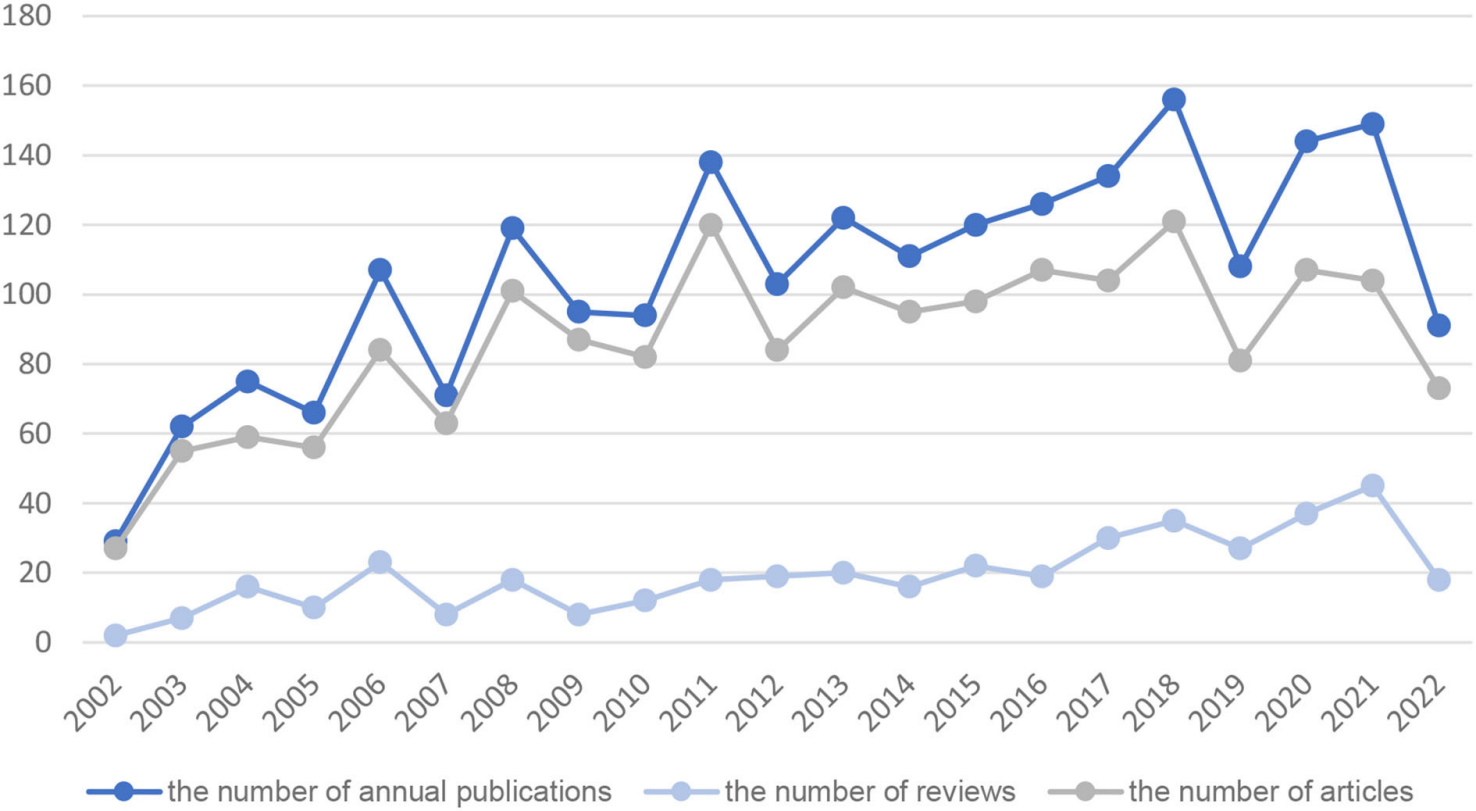
Number of publications investigating FD by year over the past 20 years.

A total of 3,532 publications were retrieved; after removing four duplicate documents, we read the titles and abstracts of each article one by one, and finally, 2,220 articles met the inclusion criteria (Figure 1). There were 1,810 (81.53%) articles and 410 (18.47%) reviews among the 2,220 documents of FD. The annual volume of productive is shown in Figure 2. The number of publications showed an overall increasing trend, but fluctuated from year to year. The steady increase in the number of publications, since 2014 with the exception of 2019, indicates that FD is of increasing interest to the research field.

### Authors

**Figure 3:**
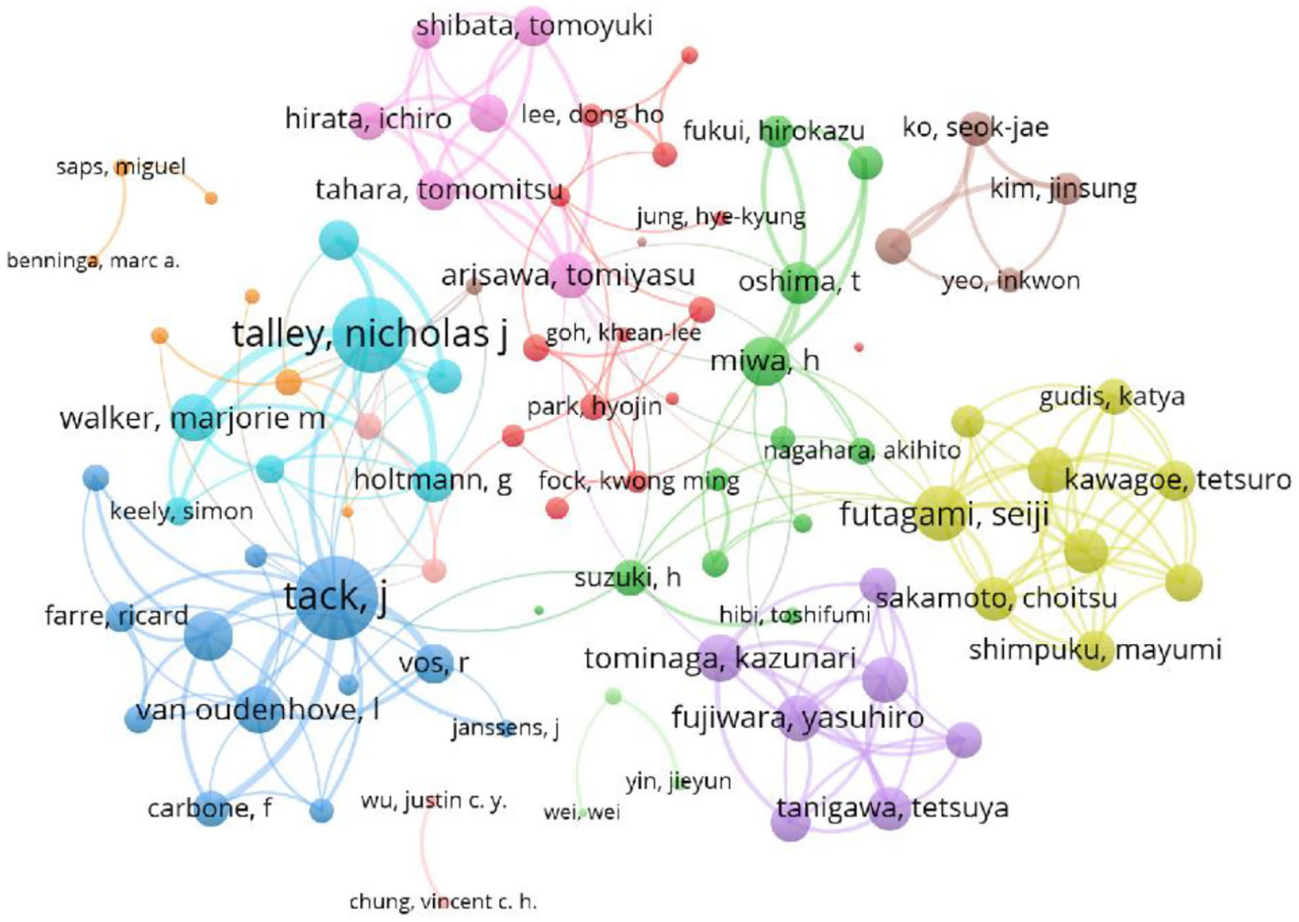
Co-occurrence of authors on FD. The size of circles was citations. The thickness of the lines indicates the strength of the relationship. The color means different cooperative networks.

Generally speaking, author co-occurrence analysis is helpful to identify the most influential authors and evaluate the cooperation ability between different teams. The author's collaboration network map is displayed in Figure 3. As shown in Figure 3, we can see that several stable collaborative teams have been formed in the field of FD research and that the teams work closely together.

A total of 8,787 authors were involved in the field of FD with 2,220 publications. The top 10 productive authors' detailed information is shown in Table 1, including the number of documents and the total link strength. Tack J from Belgium has published 171 documents ranked first and collaborated closely with other scholars. As a professor at Katholieke Universiteit Leuven, he and his colleagues Van Oudenhove L and Vanuytsel T had been deep in the field for a long time. The second highest ranking was Talley NJ with 167 articles. Their teams work closely with each other.

**Table 1:** Top 10 authors about FD.

**Rank**	**Authors**	**Organizations**	**Country**	**Documents**	**Total link strength**
1	Tack J	Katholieke Universiteit Leuven	Belgium	171	403
2	Talley NJ	University of Newcastle	Australia	167	323
4	Camilleri M	Mayo Clinic	United States	50	91
3	Holtmann G	University of Queensland	Australia	48	94
5	Walker MM	University of Newcastle	Australia	44	114
6	Zinsmeister AR	Mayo Clinic	United States	42	127
7	Van Oudenhove L	Katholieke Universiteit Leuven	Belgium	41	132
8	Miwa H	Hyogo College of Medicine	Japan	40	181
9	Vanuytsel T	Katholieke Universiteit Leuven	Belgium	34	131
10	Zeng F	Chengdu University of Traditional Chinese Medicine	China	32	127

### Organizations

The co-occurrence of organizations facilitates the understanding of partner organizations and the identification of core teams. The top 10 productive organizations' detailed information is shown in Table 2, including the number of documents, citations, and the total link strength. The top 10 prolific organizations included two Belgian organizations, four Australian organizations, and one organization from China, the United States, Canada, and Japan. The Katholieke Universiteit Leuven from Belgium has published a total of 131 articles at most, which are closely related to University Hospital Leuven. Mayo Clinic from the United States has published 127 articles, ranking second. It is a comprehensive medical center with a long history and has in-depth research in the field of gastroenterology. The University of Newcastle from Australia has the strongest overall link strength and is closely related to Katholieke Universiteit Leuven and Mayo Clinic.

**Table 2:** Top 10 organizations about FD.

**Rank**	**Organizations**	**Country**	**Documents**	**Citations**	**Total link strength**
1	Katholieke Universiteit Leuven	Belgium	131	6,060	137
2	Mayo Clinic	United States	127	8,345	205
3	University of Newcastle	Australia	91	4,321	230
4	Chengdu University of Traditional Chinese Medicine	China	44	785	80
5	Hyogo College of Medicine	Japan	39	1,021	89
6	McMaster University	Canada	36	3,580	86
7	University of Sydney	Australia	35	2,983	78
10	University Hospitals Leuven	Belgium	35	2,461	69
8	Macquarie University	Australia	34	1,219	91
9	University of Adelaide	Australia	33	1,749	71

**Figure 4:**
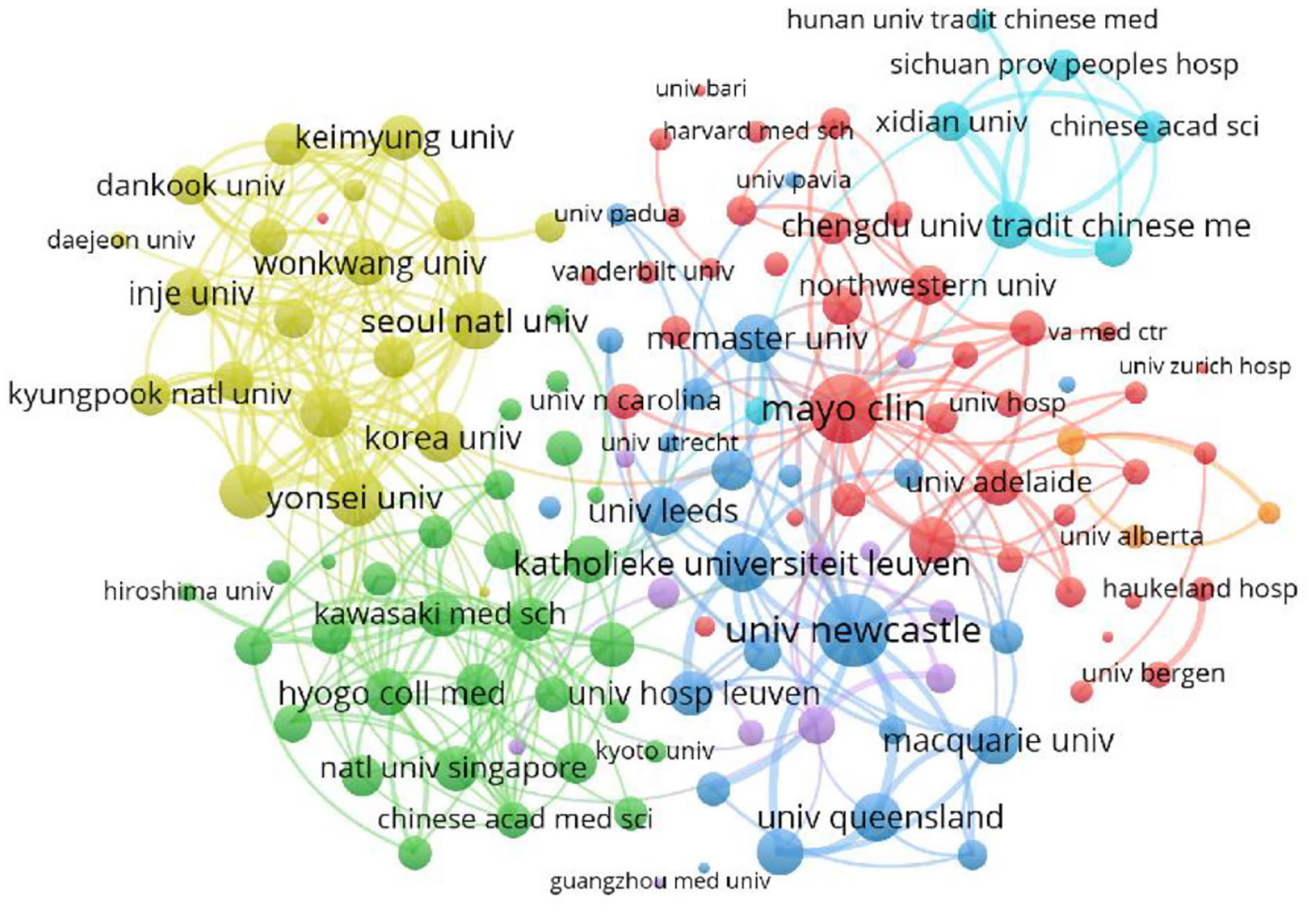
Co-occurrence of organizations on FD. The size of circles was total link strength. The thickness of the lines indicates the strength of the relationship. The color means different cooperative networks.

According to VOSviewer analysis, 2,220 documents were published by 2,236 different organizations and 139 met the threshold (minimum number of documents of an organization: 8). After excluding disjointed organizations, the remaining 139 organizations were used for the visualization map. The organizations' collaboration network map is displayed in Figure 4. As shown in Figure 4, several stable collaborative teams have been formed in the field of FD research. The three most productive organizations, represented by Katholieke Universiteit Leuven, Mayo Clinic, and the University of Newcastle, work closely together in the field of FD research.

### Countries/regions

**Figure 5:**
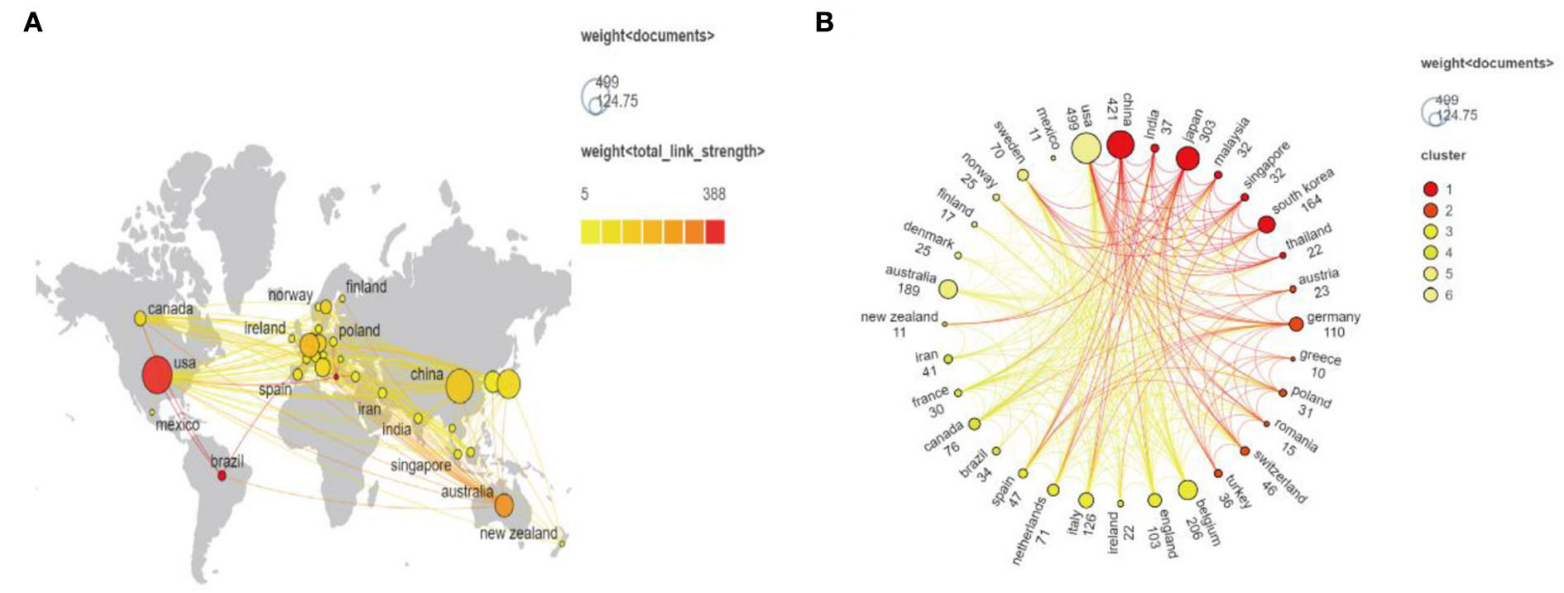
Co-occurrence of countries on FD. **(A)** A world map showing the number of publications and the strength of collaborations among countries. **(B)** A circle diagram that evaluates international collaboration between clusters.

According to the VOSviewer analysis, a total of 75 countries have published literature on FD, with the top three countries in terms of number of publications being the United States (*n* = 499), China (*n* = 421), and Japan (*n* = 303). The top 10 productive countries' detailed information is shown in Table 3, among which the United States, Belgium, and Australia rank the top three in citations, followed by China and Japan. China is the only developing country among the top 10 countries with the number of publications. It is remarkable that although South Korea ranks sixth in the number of publications, its citations and total link strength are lower than those of other countries. Figure 5A depicts national interactions involved with FD, whereas various clusters of the country's cooperation on FD were displayed in Figure 5B. Cluster analysis shows that the top 32 countries have formed six close cooperation networks, among which the United States, Belgium, and Australia have the most frequent cooperation.

**Table 3:** Top 10 countries about FD.

**Rank**	**Country/region**	**Documents**	**Citations**	**Total link strength**
1	United States	499	24,926	398
2	China	421	9,513	162
3	Japan	303	8,817	104
4	Belgium	206	12,552	191
5	Australia	189	11,442	272
6	South Korea	164	2,677	61
7	Italy	126	6,921	98
8	Germany	110	5,067	129
9	England	103	6,373	162
10	Canada	76	6,482	112

### Published journals

By the data analysis, the documents of FD from 2002 to 2022 were mainly distributed in different journals and the top 10 journals are listed in Table 4. The most prolific journal was the Neurogastroenterology and Motility with 218 documents. The 2021 impact factor of these journals ranged from 2.650 to 33.883, which is quite different; among them, Gastroenterology was the highest, and Evidence-based Complementary and Alternative Medicine was the lowest. As can be seen from Table 4, Q1 was 40%, Q2 was 40%, and Q3 was 20% in this ranking. It is helpful to find out the core journal by analyzing the distribution of publication sources and the number of published articles. Judging by the number of publications and impact factors, the Neurogastroenterology and Motility may be the most popular journal.

**Table 4:** Top 10 journals of publications about FD.

**Rank**	**Journals**	**Documents**	**2021 impact factor**	**2021 JCR partition**
1	Neurogastroenterology and motility	218	3.960	Q2
2	Alimentary pharmacology and therapeutics	101	9.524	Q1
3	Journal of gastroenterology and hepatology	90	4.369	Q2
4	Journal of neurogastroenterology and motility	80	4.725	Q2
5	American Journal of gastroenterology	79	12.045	Q1
6	World Journal of gastroenterology	73	5.374	Q2
7	Digestive diseases and sciences	68	3.487	Q3
8	Gut	46	31.793	Q1
9	Evidence-based complementary and alternative medicine	42	2.650	Q3
10	Gastroenterology	42	33.883	Q1

### Citations

Highly cited literature can reflect the research focus and trend. To some extent, the higher the number of citations, the better the quality. The top 10 high citations are listed in Table 5, and the range of citations was from 396 to 1,181. “Functional gastroduodenal disorders” (2006) had 1,181 citations; “Childhood Functional Gastrointestinal Disorders: Child/Adolescent,” was published by Rasquin A, which was with 1,073 citations; and the third was 942 citations, “The Serotonin Signaling System: From Basic Understanding to Drug Development for Functional GI Disorders” (2007) (Table 5). In the top 10 high citations, reviews and articles account for half each other.

**Table 5:** Top 10 citation analysis of documents about FD.

**Rank**	**Title**	**First author**	**Journal**	**Publication year**	**Total citations**
1	Functional gastroduodenal disorders	Tack J	Gastroenterology	2006	1,181
2	Childhood functional gastrointestinal disorders: child/adolescent	Rasquin A	Gastroenterology	2006	1,073
3	The serotonin signaling system: from basic understanding to drug development for functional GI disorders	Gershon MD	Gastroenterology	2007	942
4	American college of gastroenterology guideline on the management of helicobacter pylori infection	Chey WD	Am J Gastroenterol	2007	842
5	Kyoto global consensus report on Helicobacter pylori gastritis	Sugano K	Gut	2015	682
6	Gastroduodenal disorders	Stanghellini V	Gastroenterology	2016	593
7	Medically unexplained physical symptoms, anxiety, and depression A meta-analytic review	Henningsen P	Psychosom Med	2003	560
8	Intestinal microbiota in functional bowel disorders: a Rome foundation report	Simrén M	Gut	2013	546
9	Childhood functional gastrointestinal disorders: Child/Adolescent	Hyams JS	Gastroenterology	2016	414
10	Consensus recommendations for gastric emptying scintigraphy: a joint report of the American Neurogastroenterology and Motility Society and the Society of Nuclear Medicine	Abell TL	Am J Gastroenterol	2008	396

To analyze the citations of documents, co-citation analysis of cited references was performed. Table 6 summarizes the primary information of the top 10 most commonly cited references, including six articles and four reviews. It should be noted that among the top 10 highly cited references, eight were published in gastroenterology and one in the gut, and the quality of the literature is generally high. As can be seen from Table 6, the most frequently cited reference is Functional Gastroduodenal Disorders, which was consistent with the previous citation analysis of documents in Table 5, indicating that this article was the most acknowledged document about FD. Notably, four of the top 10 highly cited references were published by Tack J.

**Table 6:** Top 10 co-citation of cited reference about FD.

**Rank**	**Title**	**First author**	**Journal**	**Publication year**	**Total citations**
1	Functional gastroduodenal disorders	Tack J	Gastroenterology	2006	602
2	Role of impaired gastric accommodation to a meal in functional dyspepsia	Tack J	Gastroenterology	1998	367
3	Functional gastroduodenal disorders	Talley NJ	Gut	1999	329
4	Gastroduodenal disorders	Stanghellini V	Gastroenterology	2016	257
5	symptoms associated with hypersensitivity to gastric distention in functional dyspepsia	Tack J	Gastroenterology	2001	241
6	The functional gastrointestinal disorders and the Rome III process	Drossman DA	Gastroenterology	2006	223
7	Risk indicators of delayed gastric emptying of solids in patients with functional dyspepsia	Stanghellini V	Gastroenterology	1996	206
8	Pathophysiology and treatment of functional dyspepsia	Tack J	Gastroenterology	2004	196
9	Symptoms associated with impaired gastric emptying of solids and liquids in functional dyspepsia	Sarnelli G	Am J Gastroenterol	2003	187
10	Dyspepsia and dyspepsia subgroups: a population-based study	Talley NJ	Gastroenterology	1992	158

### Keywords

**Figure 6:**
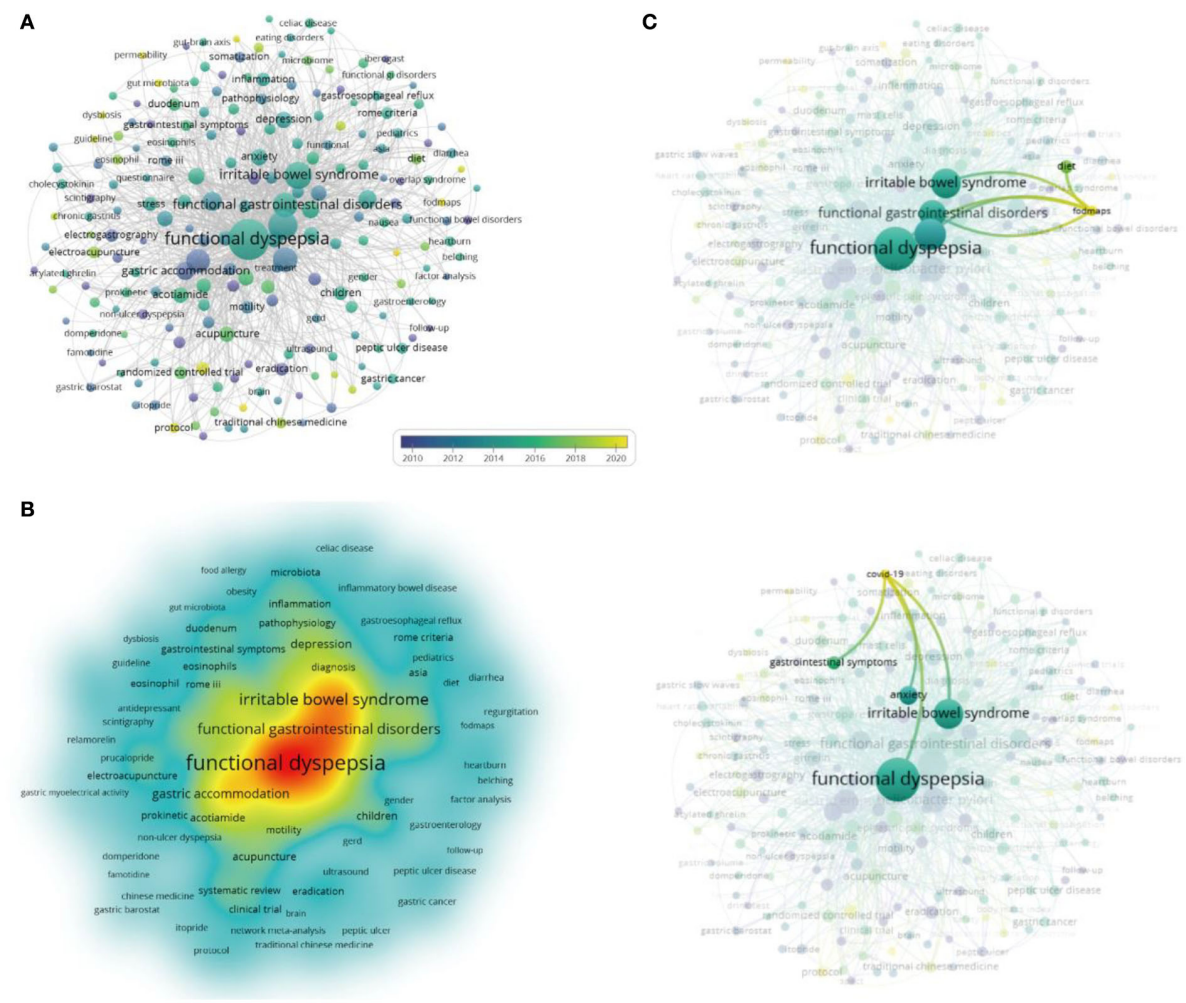
Co-occurrence of keywords on FD. **(A)** Time zone diagram of keywords changing with time. **(B)** Hot map of keywords research, the hotter the color, the stronger the frequency. **(C)** The latest progress in FD research, such as FODMAPS and COVID-19.

Analyzing the keywords of the documents can help us quickly grasp the main points of the literature and understand the development trend. Keywords from the 2,220 publications assessed in the present study were analyzed using VOSviewer (Figure 6). Table 7 shows the occurrences, average publication year, and average citation numbers of the 20 keywords. The top three keywords according to the weight of occurrences were functional dyspepsia (*n* = 836), dyspepsia (*n* = 301), and irritable bowel syndrome (*n* = 237), suggesting that FD is often studied together with different functional gastrointestinal diseases, such as irritable bowel syndrome (IBS). As can be seen from the Table 7, the average publication year of acupuncture and postprandial distress syndrome is 2016, and the average publication year of other keywords is < 2016. FD is a non-organic disease, and its relationship with anxiety, depression, and other emotions has gradually attracted the attention of the research field. The keywords were color-coded by VOSviewer based on the average publication year as shown in Figure 6A. Figure 6B shows the current research hotspots of the keywords, and the hotter the color indicates the higher frequency of occurrence. As time passed on, non-drug treatment methods such as acupuncture, probiotics, and FODMAPS, as complementary and alternative therapies, have gradually entered the program of treating FD. FODMAP diet has a therapeutic effect on functional gastrointestinal diseases, such as FD and IBS (Figure 6C). The epidemic situation in COVID-19 caused anxiety and gastrointestinal discomfort, which indirectly led to an increase in the prevalence of FD and IBS (Figure 6C).

**Table 7:** Occurrence times, average publication years, and average citation times of the top 20 keywords.

**Rank**	**Keywords**	**Weight**	**Score**	**Score**
		**(Occurrences)**	**(Avg.pub. year)**	**(Avg. citations)**
1	Functional dyspepsia	836	2014.52	18.81
2	Dyspepsia	301	2013.72	20.84
3	Irritable bowel syndrome	237	2014.48	27.06
4	Functional gastrointestinal disorders	184	2014.43	22.24
5	Helicobacter pylori	165	2011.82	20.12
6	Gastric emptying	155	2011.03	25.26
7	Gastric motility	82	2012.84	21.70
8	Gastric accommodation	71	2010.90	26.38
9	Epidemiology	66	2014.30	30.18
10	Abdominal pain	64	2015.01	32.91
11	Gastroparesis	54	2014.90	21.81
12	Anxiety	50	2014.06	27.26
13	Quality of life	48	2013.75	17.56
14	Depression	47	2013.74	32.87
15	Postprandial distress syndrome	43	2016.35	11.81
16	Acupuncture	41	2016.80	13.10
17	Constipation	41	2012.32	31.68
18	Acotiamide	40	2015.80	18.35
19	Gastroesophageal reflux disease	40	2014.40	21.65
20	Ghrelin	39	2013.13	26.54

## Discussion

### Summary

In the context of COVID-19, many people suffer from anxiety and depression due to increased work and life stress, which in turn leads to symptoms of functional indigestion, such as early satiety and postprandial fullness (16–18). As an indolent, non-organic disease, FD has attracted continuous attention in the field of gastrointestinal research. A summary of the development trend of FD is helpful for researchers to understand the development status and hotspots in recent years and guide future research. Our quantitative analysis of FD research literature published over the past 20 years found that the overall publication volume fluctuated upward during this period. According to the number of published literature, the most represented research areas were gastroenterology and hepatology, neuroscience and neurology, and general internal medicine. Among them, Tack J bridges published the most articles, and his institution, Katholieke Universiteit Leuven, is also the organization with the largest number of literature. The research field has formed a stable research team headed by Tack J and Talley NJ, and each team works closely together to promote the development of FD-related research (6, 19, 20). As one of the most influential medical organizations in the world, Mayo Clinic has in-depth research in the field of functional gastroenterology. It has the second highest number of publications but the highest frequency of citations. According to the total link strength, the University of Newcastle surpassed Katholieke Universiteit Leuven and Mayo Clinic to become the organization with the strongest total link strength, which shows that it has more cooperation and stronger ties with other organizations. Currently, several stable cooperation teams have been formed among various organizations, and there are close exchanges between different organizations.

In terms of national publications, most of the documents with large publications and high quality are concentrated in developed countries, especially in the United States, Australia, and Belgium, which may be related to their advanced medical technology and their concern for physical and mental health. At the same time, close cooperation among different countries in the world has promoted the sharing of technical knowledge and the development of FD research. As the only developing country among the top 10 most-published countries, China ranks second in the number of publications, but still lacks the emergence of several core authors, and the overall number of published authors is relatively scattered. Except for Evidence-based Complementary and Alternative Medicine, the top 10 journals are all specialized journals of gastroenterology. The 2021 journal impact factors of the top 10 journals by publication range from 2.650 to 33.883, with 80% in JCR partition 1 and 2.

### Knowledge base

Highly cited literature reflects the research hotspots in the field, and the analysis of cited references provides insight into the research base of the field. Three reports and one guideline were among the top 10 highly cited literature, indicating the importance of reports or guidelines in guiding FD-related research. At the same time, the 10 most frequently cited references in this field are listed, of which six are articles and four are reviews, all published in the ten most-published journals. As one of the four functional gastroduodenal disorders, FD has a high prevalence and affects a wide age range (21). Gender, whether or not they smoke, and whether or not they suffer from depression are all influential factors in triggering their symptoms (22–24). FD has been a major concern in the field of functional gastrointestinal disorders because of its serious impact on the quality of life and physical and mental health of patients. At present, the pathogenesis of FD is still unclear, which affects the formulation of its clinical treatment plan. Gastric motility disorders and visceral hypersensitivity are important research contents, and the changes of various substances in duodenum have also attracted the attention of the research field. Current research shows that the duodenum plays an important role in regulating gastrointestinal motility. Duodenal disorders may affect changes in neuronal signaling pathways that affect gut–brain interactions, and impaired mucosal integrity, low-grade inflammation, or increased eosinophilia may be associated with FD symptoms (6, 25, 26). Researchers are committed to exploring the pathogenesis of FD to find the most appropriate treatment plan to guide clinical practice. Currently, pharmacological treatment with proton pump inhibitors, such as omeprazole, is considered to be an effective treatment for FD, which can inhibit gastric acid secretion to protect the gastric mucosa and thus relieve the discomfort (27–29). It is worth noting that the side effects of the drug still need to be further explored. In addition, other methods are still in the exploratory stage and no consensus has been reached.

An analysis of the highly cited literature and the cited references shows that the mechanistic study of FD remains the focus of current research in the field. The development of effective treatment protocols is severely limited by the fact that the FD mechanism is still unclear. Only when we have a clear understanding of its mechanism, we can formulate a targeted treatment plan, effectively relieve discomfort symptoms, and achieve a cure effect. In future, with the gradual clarification of FD pathophysiology, it may be possible to develop personalized treatment plans for different disease symptoms and staging to achieve precise treatment to better relieve patients' discomfort and improve their quality of life.

### Research hotpots and trends

By analyzing the keywords, we can quickly understand the key content of the literature and the research trends in a specific research field. In this study, the top 20 most frequently occurring keywords are listed, and the network co-occurrence-based keywords reveal the research trend of FD and provide information on the types of research in this field. In this study, we identified two categories of research on FD: basic research exploring the pathogenesis of FD and monitoring of corresponding indicators, and clinical research investigating the efficacy of FD on specific treatment regimens. Gastroesophageal reflux disease (GERD) and irritable bowel syndrome (IBS) are two gastrointestinal disorders that affect a large portion of the general population, with important implications for quality of life and healthcare costs (30, 31). There is extensive overlap between IBS, FD, and GERD reported in the literature, and the pathophysiological mechanism between them still needs to be further explored (32). The high frequency of gastric motility, gastric emptying, and gastric accommodation in the top 20 keywords may be related to the continuous and in-depth study of gastrointestinal motility disorders in the pathogenesis of FD (33–35). Gastrointestinal motility dysfunction is recognized as one of the potential pathogeneses of FD, mainly including abnormal migrating motor complex (MMC), abnormal gastric electrical activity, abnormal receptive relaxation, and gastric emptying disorder. MMC is the main movement form of stomach and small intestine in the interdigestive period, which is characterized by periodic appearance and spontaneous circulation. It is divided into four periods, with the third period in particular being the most important. A study shows that MMC of FD patients is abnormal during digestion, that is, migrating and phase disorder, which does not normally remove bacteria and contents in intestinal tract, and aggravate the symptoms of indigestion and abdominal pain (36). Further exploration of the mechanism of gastrointestinal motility disorder will help us to deeply understand the pathophysiology of FD and develop targeted measures for treatment. Furthermore, the recruitment of inflammatory cells, such as eosinophils, and alterations in mucosal integrity were demonstrated in FD patients.

The persistence of functional gastrointestinal disorders places a heavy burden on the healthcare system (37). According to the Rome IV, functional gastrointestinal disorders are defined as disorders of gut–brain interactions, where the biological basis of gut–brain interactions is the bidirectional dynamic communication between the gastrointestinal tract and the brain *via* neurotransmitters (38). At present, the research on the molecular level of the pathogenesis of FD is gradually deepening. On the one hand, the disorder of the immune system and gastrointestinal microbiome will be transmitted to the central nervous system through the gut–brain axis pathway and then cause various physiological changes; on the other hand, psychological, social, and mental factors react on the nervous system and affect the secretion of hormones in the gastrointestinal tract through the gut–brain axis. Neurotransmitters such as ghrelin, VIP, and SP are distributed in the brain and gastrointestinal tract and share the same receptors (39, 40). Their roles in gut–brain interactions have gradually attracted the attention of the medical community, but their specific targets have not yet been identified clearly. In addition, a study found that patients with different psychosocial characteristics have different disease courses and treatment effects (41). Compared with healthy people, FD patients have higher emotional and physical disorders, and the prevalence of anxiety, depression, and neuroticism is also significantly increased (42, 43). Under the background of COVID-19, psychological problems such as anxiety and depression have increased with the increasing stress in people's lives, further leading to the development of gastrointestinal discomfort symptoms, such as early satiety (44). At the same time, the change of psychological state also leads to the increase of the incidence of FD and aggravates the discomfort symptoms of FD patients (45). Clinically, the use of antipsychotics and tricyclic antidepressants can significantly improve symptoms in patients with FD compared with placebo (46). This further indicates the important role of psychological factors in the pathogenesis of FD and also suggests that psychotropic drugs may be an effective method for the treatment of FD.

The complex pathogenesis of FD has not yet been fully elucidated. Commonly used in the clinical treatment are gastrointestinal motility drugs, antacids, and antidepressants. Although they have certain curative effects, they all have different degrees of side effects, and the recurrence rate after drug withdrawal is high (7). Due to the limited efficacy of traditional pharmacological drugs, physicians and patients may seek other medical and non-medical solutions to the problem (47). It is worth noting that complementary replacement therapy has a significant effect on long-term improvement of discomfort symptoms and quality of life in patients with FD and has gradually entered the field of clinical treatment. Food often causes uncomfortable symptoms in FD patients, but the exact mechanism is not fully understood and may be related to microbial changes in the gastrointestinal tract due to food intake (48). Studies have explored the efficacy of low fermentable oligosaccharides, disaccharides, monosaccharides, and polyols (FODMAP) diet and traditional diets in FD patients, and the results have shown that both diets can improve symptoms and improve the quality of life in patients (49). In addition, patients with postprandial distress syndrome or bloating were better treated with the FODMAP diet. However, the latest guidelines of the British Society of Gastroenterology on FD pointed out that there is currently insufficient evidence to support dietary therapy (28). In future research, we should try our best to carry out large-scale RCT experiments of FD diet therapy and explore personalized dietary treatment plans, so as to obtain reliable and real data from large samples to provide guidance for clinical treatment. In addition, traditional Chinese medicine techniques such as acupuncture and electro-acupuncture also play an important role in the treatment of FD (50). However, there is no unified conclusion on the mechanism of acupuncture in the treatment of FD, which may be related to the regulation of cerebral nerve-related pathways through gut–brain pumping and then regulating the secretion of gastrointestinal hormones (51, 52). It is a promising complementary and alternative therapy for the treatment of postprandial distress syndrome, as it is effective in relieving major discomfort symptoms and has a long-lasting effect (53). At present, the systematic reviews and meta-analyses of acupuncture treatment of FD are increasing year by year, but the quality is generally low, which affects the international promotion and application of acupuncture (54). In conclusion, FD has a long onset and a high recurrence rate, and long-term use of pharmacological treatment can cause certain problems for the physical and mental health of patients. In future, the combination of non-pharmacological treatment and pharmacological treatment for FD may become the main development direction.

### Strengths and limitations

As a gastrointestinal disease that plagues many people, FD has gradually developed into a chronic disease, bringing a heavy burden to the medical and health field. Since the Rome I standard was put forward, the related research on FD has also experienced rapid growth, but no research has comprehensively analyzed the current situation and development trend of FD. This study is the first to use the method of bibliometrics to analyze the development, changes, and research hotspots of FD in the past 20 years, to provide research reference for peers. Although this study provides a detailed analysis of the FD research field, there are some limitations. First, this study only searched the WOS Core Collection, ignoring databases such as Embase and Medline; however, these databases contain a large amount of literature. This may lead to incompleteness of the included literature. Second, we limit the language of the research to English and the type of research to reviews or articles, which may lead to the omission of some high-quality documents. Third, when conducting author analysis, since the same author may belong to different organizations in different periods, this study presents the organization to which the latest author belongs, which may lead to a mismatch between the number of published articles by the organization and the author. Finally, this study took a long time from start to finish, and literature published during this period could not be included.

## Conclusion

FD, as one of the most common functional gastrointestinal diseases, seriously affects people's quality of life and places a heavy burden on the healthcare system. As modern people gradually pay more attention to their health, it is urgent to seek effective FD treatment options. This study is the first comprehensive analysis of the FD research field using bibliometric methods. Our findings are helpful to comprehensively review the research history of FD, understand its current research progress and future development trend, and provide reference for researchers in this field to further study.

## Data availability statement

The original contributions presented in the study are included in the article/supplementary material, further inquiries can be directed to the corresponding author.

## Author contributions

XW: design this research, write the first draft, and revise the language and characters. HL and WL: reviewing the literature and analyzing. HX: rechecking the manuscript and putting forward suggestions for amendment. All authors contributed to the article and approved the submitted version.

## Funding

This study was supported by the National Natural Science Foundation of China with Grant No. 81403312.

## Conflict of interest

The authors declare that the research was conducted in the absence of any commercial or financial relationships that could be construed as a potential conflict of interest.

## Publisher's note

All claims expressed in this article are solely those of the authors and do not necessarily represent those of their affiliated organizations, or those of the publisher, the editors and the reviewers. Any product that may be evaluated in this article, or claim that may be made by its manufacturer, is not guaranteed or endorsed by the publisher.
